# A Treatise on Practical Anatomy

**Published:** 1892-06

**Authors:** 


*A Treatise on Practical Anatomy.* By HENRY C. BOENNING, M.D. Pp. xvi., 481. Philadelphia: F. A. DAVIS. 1891.

This book has been written especially for students of anatomy and surgery, and the aim of the author has been to arrange the subject so as to make it equally serviceable as a text-book and a dissector. There are now so many well-established text-books on anatomy that an author in bringing forward a new work on the subject ought either to show some new arrangement of material, or else to illustrate it by original diagrams.

The book before us may have some claims on the former ground, inasmuch as the muscles, arteries, and nerves are arranged in the form of very brief tables. This method may enable some students to learn rapidly the main facts, but this must necessarily be at the expense of accuracy and detail. Thus, to take an example, the extensor communis digitorum muscle of the forearm is described in the following words: "Origin—External epicondyle and intermuscular septa. Insertion—Divides into four tendons, which are inserted on the dorsal and lateral surfaces of the first, second, and third phalanges." This description may serve to recall the muscle to a student who has once properly learned it, but would be of little use to one who was dissecting the muscle for the first time, and wished to read an account of it.

The plates are nearly all copied from other well-known works, and many of them are so cloudy and indistinct that it is difficult to understand them. The book is printed in large type, with good broad margins, and is apparently an attempt to make the anatomy of the human subject easy reading.

